# Evaluation of vegetable and fish oils diets for the amelioration of diabetes side effects

**DOI:** 10.1186/2251-6581-12-13

**Published:** 2013-02-21

**Authors:** Nadia Saleh Al-Amoudi, Huda A Abu Araki

**Affiliations:** 1 Nutrition and Food Department, King Abdulaziz University, P. O. Box 3108, Jeddah, 23435 Saudi Arabia; 2 Laboratory Animals Unit, King Fahad Medical Research Center, P. O. Box 80216, Jeddah, 21589 Saudi Arabia

**Keywords:** Vegetable oils, Fish oils, Diets, Diabetes cod liver oil, Sesame oil, Lipid profile, Liver function, Kidney function

## Abstract

**Background:**

In the existing literature, the evidence regarding the effects of certain oils on the amelioration of hyperglycemia contains ambiguities and contradictions; and with regard to other oils, the quantity of existing studies is scant.

**Objective:**

To assess the influence of sesame, garden rocket, organic olive, thyme, fenugreek, hazelnut, and cod liver oil on serum glucose, liver function, and kidney functions.

**Methods:**

Male albino rats were injected with streptozotocin (60 mg/kg BW). The duration of the experiment was 28 days. Maximum recovery of occurred wasting attributable to diabetes was found in the sesame and cod liver groups.

**Results:**

With respect to ameliorating and/or preventing the side effects of diabetes on liver function, this experiment showed that thyme, organic olive, cod liver, and fenugreek oils were efficacious. Turning to serum lipid profile, organic olive oil not only ameliorated but also prevented the changes of TC, HDL, LDL, and AI. Vegetable and cod liver oil diets resulted in a marked amelioration of renal dysfunction, but they were unable to prevent this side effect. Similar, oil diets were unable to mask the increase in serum glucose due to diabetes mellitus.

**Conclusion:**

On the basis of these findings, it could be recommended that when attempting oil diet treatment for the side effects of diabetes, a blend of the various specific treatments which showed best results should be employed in order to achieve improvement with respect to all parameters; and in part, this is because a synergism between the various treatments can be expected.

## Introduction

The term diabetes mellitus refers to a group of metabolic disorders, all of which share one common manifestation: hyperglycemia – that is, high level of blood sugar. Chronic hyperglycemia eventually has severely detrimental effects on the eyes, kidneys, nerves, heart, and blood vessels. Further, diabetes mellitus has serious side effects, which consequently provide openings for opportunity diseases [1]. Conversely, insulin resistance can occur as a secondary condition of various diseases, including bacterial infection, hypothyroidism, dioestrus, hyperdienocorticism, and renal, hepatic, or cardiac inefficiency. The etiology of diabetes mellitus can be traced to the absence of insulin action, because in the absence of this action, sugar cannot be converted into energy [2, 3].

In 1997, an estimated 124 million people worldwide had diabetes; and 97% of these people had type 2 diabetes, otherwise known as non-insulin dependent diabetes mellitus (NIDDM) or adult-onset diabetes. In the contemporary sociocultural context, type 2 diabetes is consistently on the rise due to the rampant proliferation of the “obesity epidemic” [4]. Presently, 17% of people older than 65 years are afflicted by this form of diabetes [5]. Further, the World Health Organization [6] has reported that al least 171 million people worldwide suffer from diabetes; this number constitutes 2.8% of the world population. The prevalence of the condition is clearly increasing, and it has been estimated that by the year 2030, this percent will have almost doubled, nearly reaching 5.6%. Diabetes mellitus occurs throughout the world, but it is more common – especially the type 2 version – in the more developed countries. That said, though, the greatest rise in prevalence in the near future is expected to be in Asia and Africa [7]. Recently, the number of people worldwide afflicted by diabetes has been estimated at 217 million; and the total economic burden of type 2 diabetes has been estimated to be on the order of 8% of the total health expenditures of the United States [8, 9].

Since ancient times, plants and herbal preparations have been used for various folk medicinal purposes, including the treatment of glycemia [10]. Some oils, and in particular olive oil, have been claimed as being highly efficacious in the treatment of type 2 diabetes mellitus [11]. On the other hand, though, [12] have reported that very little is known about the effect of olive oil on type 2 diabetes patients, even though virgin olive oil has demonstrable efficacy in protecting LDL from oxidation and in restoring the levels of dihomo-gamma-linolenic acid (20: 3, n – 6) in serum cholesterol esters, and phospholipids in diabetic patients; this is attributable to the effect of monounsaturated fatty acids and presence of antioxidants in the oil. Further, several investigators have recommended the use of dietary supplementation with fish oil as a means of treating diabetes [13, 14]. Likewise, [15] have concluded that fish oil diet treatment for type 2 diabetes have positive effects on triglyceride and LDL cholesterol levels. With respect to other vegetable and animal oils, there is a considerable dearth of sufficient research into their efficacy in ameliorating the health problems generating by the condition of diabetes. Accordingly, the present study deals with the evaluation of sesame oil, garden rocket oil, olive oil, thyme oil, fenugreek oil, hazelnut oil, and cod liver oil on serum glucose and liver function as well as renal functions, using albino rats as the test subjects.

## Materials and methods

A total of 45 male albino rats, weighing 150 +/− 5 grams each and from the Wistar strain, were obtained from the animal care house in Pharmacy College at King Saud University and transferred to King Fahad Medical Research Center in Jeddah. A total of 40 male albino rats were injecting with streptozotocin (60 mg/kg BW) twice a day for two weeks in order to induce diabetes [16]. All animal procedures were performed according to the Guide for The Care and Use of Laboratory Animals of the National Institute of Health [17] and Canadian Council on Animal Care [18], and approved by Research ethics committee of King Abdulaziz University. (Reference No 1005-13).Cod (Gadus callarias) liver oil was purchased from the pharmacy while the vegetable oils (sesame – Sesamum indicum; garden rocket – Eruca sativa; organic olive oil – Olea eurepaea; thyme – Thymus vulgaris; fenugreek – Trigonella foenum graecum; and hazelnut – Corylus americana) were obtained from the local market of Jeddah. Each oil was used in its raw state, without any preparation to make it resemble the form in which it is consumed by the average person.

In this experiment, 5 rats were assigned to each of 9 groups. There were 2 control groups: the control (-) group consisted of rats which were apparently healthy and fed diet with corn oil, while the control (+) group consisted of rats with diabetes which were provided with the same diet as the control (-). The remaining 7 groups corresponded to the 7 different kinds of oils being tested. Each oil was added to the animals’ diet at 10% level for 28 days. The control group (-) and (+) were fed on a basal diet [19]. The diets were obtained from Dyets Company – American (modified soy-based AIN-93 purified rodent diet to meet 1955 NRC requirements without soybean oil powder). The adaptation diet consisted of 40 g/kg corn oil (AIN – 94M) rodent diet for 2 weeks, and the experimental diets consisted of 100 g/kg of oils of different types for the experimental groups and both control groups for 4 weeks.

Blood samples were taken from the tail of the rats in order to ensure that diabetes had been established. During the adaptation period, the vitamin mixture used was as specified by [20], and the salt mixture used was as specified by [21]. The detailed preparation of the diets has been presented previously in [19]. The diets were AD libtum to the rats every day; 24-hour food intake was recorded on a daily basis, and body weight was recorded every second day. On the basis of these measurements, total food intake over the 4-week experimental period was calculated, along with percentage body weight gain (BWG%) and food efficiency ratio (FER).

Serum glucose (GLU) was measured according to [22]; glumatic oxalo acetic transaminases (AST) and glutamic pyruvic transaminases (ALT) were measured as described by [23]; alkaline phosphatas (ALP) was determined by the procedure delineated by [24]; AST/ALT ratio was calculated according to [25]; serum total protein (TP) was determined according to the method described by [26]; albumin (Alb) was determined as per the method of [27] modified by [28]; serum globulin (Glb) was calculated by difference (Glb = TP – Alb) according to [29] equation; triglycerides (TG) were determined after [30]; VLDL – c and LDL – c determined was carried out according to the method of [31] as follows: VLDL – c = TG/5.

LDL – c = total cholesterol (TC) – (HDL – c + VLDL – c); TC was quantified according to [32] and [30]; HDL – c was determined according to [33] and [34]; atherogenic index AI = (VLDL + LDL/HDL) was calculated according to [35]; creatinine was estimated by the method of [36]; urea nitrogen was determined according to [37]; and uric acid was estimated by the method described by [38]. The results were expressed as mean values with their standard deviation. Intergroup comparison was performed by one-way analysis of variance (ANOVA) test as described by [39]. The student’s T-test was used to compare the means; with significance set al the P < 0.05 level.

## Results and discussion

The purpose of this experiment was to investigate the effects of vegetable and fish oils on serum glucose and the possible amelioration of the side effects of diabetes mellitus, including liver and renal functions. Again, the oils used were not subjected to any of the preparations or biochemical changes which they would ordinarily have undergone by the time they were ingested by a effect of vegetable and cod liver oils on body weigt gain (BWG) of hyperglycemic rats consumer.

In the experiment Table 1, it was found that while rats afflicted with diabetes showed profound wasting – a 58.5% decrease relative to the (−) control group – the vegetable and fish oil diets were capable of reversing this change. The greatest improvement in BWG – a 31.8% relative to the (+) control group – was recording for the sesame oil and cod liver oil groups, followed by the hazelnut oil group (27.3%) and the organic olive oil group (18.2%). The lowest increase of BWG compared to the control (+) rats was found in the garden rocket, thyme, and fenugreek oil groups, with a BWG increase of 9.1% relative to the control (+) group. Even in these latter three cases, though, the observed BWG increase was tangible and significant. None of the treatments elevated the BWG to the level recorded for the healthy rats in the control (−) group, although the rats in the sesame and cod liver oil groups attained 55% of the weight recorded for the healthy control (−) rats.

**Table 1:** **Body weight gain (BWG) of diabetic rats as affected by feeding with vegetable and cod liver oils**

Oil groups	Parameter
	BWG (g/28 days)
Control (−)	53.00 ± 2.57^a^
Control (+)	22.00 ± 1.82^e^
Sesame oil	29.00 ± 1.73^b^
Garden rocket oil	24.00 ± 1.32^d^
Organic olive oil	26.00 ± 1.28^c^
Cod liver oil	29.00 ± 1.29^b^
Thyme oil	24.00 ± 0.86^d^
Fenugreek oil	24.00 ± 0.25^d^
Hazelnut oil	28.00 ± 1.37^b^
L.S.D.	1.529

Values demote means ± SD.

Mean with different letter in the same column indicate nonsignificant difference at P < 0.05 and vice versa.

### Effect of vegetable and cod liver oils on liver function of hyperglycemic rats

Data presented in Table 2 show the liver enzymes activities and AST/ALT ratio.

**Table 2:** **Liver AST, ALT, ALP enzymes activities (U/L) and the AST/ALT ratio**

Oil groups	Parameter			
	AST	ALT	ALP	AST/ALT
Control (−)	112.7 ± 2.52^e^	72.0 ± 2.65^f^	155.0 ± 5.57^i^	1.57 ± 0.02^b^
Control (+)	195.0 ± 4.85^a^	117.5 ± 3.95^a^	412.3 ± 7.51^a^	1.65 ± 0.06^a^
Sesame oil	128.3 ± 2.85^b^	103.6 ± 3.14^d^	281.0 ± 5.29^e^	1.24 ± 0.02^c^
Garden rocket oil	127.0 ± 2.65^bc^	112.0 ± 2.64^b^	360.0 ± 6.08^c^	1.13 ± 0.02^e^
Organic olive oil	124.5 ± 3.96^cd^	75.0 ± 2.57^f^	242.5 ± 6.61^g^	1.66 ± 0.03^a^
Cod liver oil	123.0 ± 2.64^d^	75.0 ± 1.73^f^	227.0 ± 6.27^h^	1.64 ± 0.03^a^
Thyme oil	106.5 ± 3.04^f^	100.0 ± 3.36^e^	270.5 ± 5.07^f^	1.07 ± 0.05^f^
Fenugreek oil	126.0 ± 3.46^bcd^	107.0 ± 2.13^c^	373.0 ± 9.94^b^	1.18 ± 0.04^d^
Hazelnut oil	126.0 ± 3.61^bcd^	106.0 ± 3.60^cd^	307.5 ± 6.54^d^	1.19 ± 0.03^d^
L.S.D.	3.192	3.127	5.667	0.031

Values demote means ± SD.

Means with different letter in the same column indicate nonsignificant difference at P < 0.05 and vice versa.

It was found that due to hyperglycemia, the AST, ALT, ALP, and AST/ALT measures increased. However, feeding the diabetic rats with diets containing vegetable and fish oils reversed these changes. Maximum reduction of these measures as compared to the control (+) group was found in the thyme oil, organic olive oil, and cod liver oil groups; and in fact, the AST, ALT, ALP an AST/ALT values for the thyme oil group were even lower than those recording for the control (−) group. This finding directly implies that this treatment strategy is highly efficacious in not only ameliorating but even curing the side effects of hyperglycemia concerning liver dysfunction.

#### Serum total protein (TP), albumin (Alb), globulin (Glob) and albumin/ globulin (Alb / Glob) ratio

Further, it was noticed that whereas hyperglycemia reduced serum TP, Alb and Alb/Glob and raised Glob, the feeding of diabetic rats on vegetable and fish oils reversed these changes as well. Different degrees of improvement occurred according to the different types of oils used (Table 3). Maximum improvement of TP, Alb, Glob, and Alb/Glob values were recorded for the organic olive and thyme oils, thyme oil, cod liver oil, and fenugreek oil, respectively. It is extremely noteworthy that the values recorded for the these treatment groups were even better than the corresponding values recorded for the control (-) group, which again indicates the remarkable efficacy of oils in mitigating and even altogether reversing the side effects of diabetes mellitus on liver function. These same conclusions were also found with respect to serum liver enzymes (Table 2).

**Table 3:** **Serum total protein (TP), albumin (Alb), globulin (Glob) and albumin/globulin (Alb/Glob) ratio**

Oil groups	Parameter			
	(TP) g/dl	(Alb) g/dl	(Glob) g/dl	(Alb/Glob) g/dl
Control (−)	81.30 ± 2.07^b^	12.80 ± 0.72^bc^	68.50 ± 2.29^c^	0.178 ± 0.003^e^
Control (+)	71.70 ± 3.66^f^	11.00 ± 0.44^d^	75.70 ± 1.59^d^	0.145 ± 0.007^e^
Sesame oil	79.70 ± 2.42^c^	13.40 ± 0.53^ab^	66.00 ± 2.18^d^	0.203 ± 0.002^b^
Garden rocket oil	81.00 ± 2.00^bc^	13.00 ± 0.17^bc^	68.00 ± 1.92^c^	0.191 ± 0.002^d^
Organic olive oil	85.30 ± 1.53^a^	12.60 ± 0.52^c^	72.70 ± 1.57^b^	0.174 ± 0.003^g^
Cod liver oil	74.30 ± 2.64^d^	12.50 ± 0.26^c^	61.80 ± 1.58^f^	0.202 ± 0.004^b^
Thyme oil	84.00 ± 1.80^a^	13.80 ± 0.27^d^	71.20 ± 1.45^b^	0.194 ± 0.003^c^
Fenugreek oil	76.00 ± 1.37^d^	12.80 ± 0.24^bc^	68.20 ± 1.19^ef^	0.209 ± 0.006^a^
Hazelnut oil	75.50 ± 1.50^de^	11.30 ± 0.61^d^	64.20 ± 1.12^e^	0.176 ± 0.003^f^
L.S.D.	1.371	0.577	1.696	0.002

Values demote means ± SD.

Means with different letter in the same column indicate nonsignificant difference at P < 0.05 and vice versa.

### Effect of vegetable and cod liver oils on serum lipids profile of diabetic rats

In addition, the data presented in Table 4 reveals that hyperglycemia cause an increase in TC, TG, LDL and AI and a significant decrease in HDL. The present study showed that the vegetable and fish oil diets had the opposite effect; once again, then, the data indicates the value of this strategy with respect to the amelioration of the side effects of diabetes mellitus. In the particular aspect under consideration at the moment, it was found that organic olive oil was most efficacious in addressing levels of TC, HDL, LDL, and AI, and that sesame oil was most efficacious in addressing TG – and consequently VLDL, which is calculated as TG/5. For all the parameters (except TG and VLDL), organic olive oil not only ameliorated but also prevented changes, and values of TC, HDL, LDL, and AI were recorded as even less than those of the control (−) group – that is, the perfectly healthy rats – after 28 days of feeding.

**Table 4:** **Effect of vegetable and cod liver oils on serum lipids profile (m mol/L) and atherogenic index (AI) of diabetic rats**

Oil groups	Parameter					
	TC	TG	VLDL	HDL	LDL	Al ratio
Control (−)	1.35 ± 0.04^f^	0.37 ± 0.01^f^	0.07 ± 0.001^f^	0.69 ± 0.02^f^	0.59 ± 0.0^g^	0.96 ± 0.06^e^
Control (+)	2.25 ± 0.07^a^	0.77 ± 0.06^a^	0.15 ± 0.010^a^	0.63 ± 0.09^h^	1.47 ± 0.09^a^	2.57 ± 0.08^a^
Sesame oil	1.91 ± 0.06^b^	0.45 ± 0.02^e^	0.09 ± 0.001^e^	0.78 ± 0.03^d^	1.04 ± 0.04^c^	1.45 ± 0.05^c^
Garden rocket oil	1.76 ± 0.02^c^	0.54 ± 0.03^d^	0.11 ± 0.010^d^	0.87 ± 0.05^b^	0.78 ± 0.03^e^	1.02 ± 0.05^d^
Organic olive oil	1.26 ± 0.03^f^	0.65 ± 0.01^b^	0.13 ± 0.020^b^	0.94 ± 0.02^a^	0.19 ± 0.02^h^	0.34 ± 0.02^g^
Cod liver oil	1.71 ± 0.03^cd^	0.61 ± 0.03^c^	0.12 ± 0.030^d/c^	0.83 ± 0.05^c^	0.76 ± 0.05^e^	1.06 ± 0.06^d^
Thyme oil	1.99 ± 0.08^b^	0.46 ± 0.02^e^	0.09 ± 0.002^e^	0.73 ± 0.03^e^	1.17 ± 0.08^b^	1.73 ± 0.06^b^
Fenugreek oil	1.60 ± 0.07^e^	0.56 ± 0.02^d^	0.11 ± 0.010^d^	0.87 ± 0.02^b^	0.62 ± 0.05^f^	0.84 ± 0.03^f^
Hazelnut oil	1.61 ± 0.02^dc^	0.45 ± 0.03^e^	0.09 ± 0.002^e^	0.66 ± 0.02^g^	0.86 ± 0.03^d^	1.44 ± 0.05^c^
L.S.D.	0.101	0.020	0.008	1.696	0.027	0.041

Values demote means ± SD.

Mean with different letter in the same column indicate nonsignificant difference at P < 0.05 and vice versa.

### Effect of vegetable and cod liver oils on renal function of diabetic rats

Moving on to other parameters, it is evident (Table 5) that hyperglycemia has profound negative effects on renal function, leading to significantly elevated levels of serum creatinine, BUN, and uric acid. The best treatment for these three parameters differed, with sesame and hazelnut oil, thyme oil, and hazelnut oil having the most significant effects on creatinine, BUN, and uric acid, respectively. While significant amelioration of side effects was detected here as well, the treatment influences weren’t quite as profound as they were with respect to the other parameters discussed above: while amelioration did occur, none of the treatments succeeded in “beating” the baseline levels of the healthy control (−) group. This may suggest that renal dysfunction is one of the more pernicious side effects of diabetes mellitus: while the oil diet treatment did bring about amelioration, they weren’t successful in prevention and outright reversal.

**Table 5:** **Effect of vegetable and cod liver oils on renal function of hyperglycemic rats**

Oil groups	Parameter		
	Creatinine μ mol/L	Blood urea nitrogen	Uric acid μ mol/L
		(BUN) m mol/L	
Control (−)	40.00 ± 0.62^e^	6.10 ± 0.17^f^	58.50 ± 0.46^e^
Control (+)	44.30 ± 0.60^a^	22.20 ± 0.72^a^	67.80 ± 0.72^a^
Sesame oil	40.70 ± 0.27^d^	19.20 ± 0.71^c^	65.30 ± 1.57^b^
Garden rocket oil	42.00 ± 0.43^c^	15.30 ± 0.61^d^	61.00 ± 1.73^c^
Organic olive oil	43.40 ± 0.58^b^	21.70 ± 0.53^a^	60.50 ± 1.50^cd^
Cod liver oil	42.00 ± 0.26^c^	19.90 ± 0.96^b^	59.50 ± 2.18^de^
Thyme oil	44.00 ± 0.30^a^	14.50 ± 0.51^e^	61.00 ± 1.14^c^
Fenugreek oil	44.00 ± 0.17^d^	15.40 ± 0.55^d^	55.70 ± 1.13^f^
Hazelnut oil	40.80 ± 0.22^d^	14.60 ± 0.26^e^	53.50 ± 1.32^g^
L.S.D.	0.552	0.678	1.305

Values demote means ± SD.

Mean with different letter in the same column indicate nonsignificant difference at P < 0.05 and vice versa.

### Effect of vegetable and cod liver oils on serum glucose of hyperglycemic rats

Finally, while hyperglycemia raised serum glucose level, this level was reduced significantly when diabetic rats were placed on diets consisting of vegetable and fish oils. The best results were reported in the sesame oil and garden rocket oil groups, with the rats in these groups showing a 57.7-59.1% decrease compared to the control (+) group. Although the oils weren’t able to provide a preventative function with respect to this parameter (the level was still higher than that of the control (−) group), the effects were still meaningful, as can be seen in the comparison with the control (+) group (Table 6).

**Table 6:** **Effect of vegetable and cod liver oils on serum glucose of hyperglycemic rats**

Oil groups	Parameter
	Glucose m mol/L
Control (−)	6.60 ± 0.06^f^
Control (+)	33.10 ± 1.88^a^
Sesame oil	14.00 ± 0.34^e^
Garden rocket oil	14.20 ± 0.72^e^
Organic olive oil	31.00 ± 1.73^b^
Cod liver oil	19.50 ± 0.53^d^
Thyme oil	27.40 ± 1.44^c^
Fenugreek oil	27.60 ± 0.53^c^
Hazelnut oil	28.80 ± 1.71^c^
L.S.D.	1.529

Values demote means ± SD.

Mean with different letter in the same column indicate nonsignificant difference at P < 0.05 and vice versa.

From these findings, two general conclusions can be drawn. First, it is quite evident that vegetable and fish oils are extremely efficacious in ameliorating the side effects of type 2 diabetes mellitus. In every single parameter, a significant effect was recorded in the experimental groups relative to the control (+) group: that is, when compared to diabetic rats on a normal diet, the diabetic rats on the oil diets experienced markedly improved health. Furthermore, in several of the parameters, the quality of the levels of certain experimental groups surpassed even that of the control (−) group – or in other words, with respect to these parameters, diabetic rats on oil diets actually fared even better than completely healthy rats on normal diets. This means that not only were the experimental diets successful in ameliorating the side effects of diabetes, in certain cases they were even capable of entirely preventing those side effects.

Following from this, the second main conclusion is that not all experimental diets were equally successful in addressing all the parameters under investigation. In fact, there was a significant divergence with respect to which specific oils had particularly exemplary effects on which specific parameters. This would seem to suggest that if a diabetes treatment protocol is developed on the basis of these findings, it may be advisable to use some cocktail involving all of the various oils under consideration here. It could be expected that the various oils will interact synergistically with each other; and if each oil addresses the specific parameter which it is most efficacious at addressing, then nearly all the various parameters investigated in this study would be addressed by the treatment cocktail.

It is worth noting that the findings of this study are supporting by the findings of other studies in the extant literature on this subject. For example, El-Missiry and El-Gindy [40] conducted an experiment which consisted of the use of alloxan to induce diabetes mellitus in rats, followed by the use of oil from the seeds of the plant *Eruca saliva* in order to ameliorate the diabetes side effect of oxidative stress. The present study didn’t use either this specific catalyst for diabetes or this specific substance for amelioration; but clearly, this study was conceptually and methodologically very similar to the present study. It is therefore relevant that [40] found that their oil was effective in ameliorating the side effects of diabetes mellitus and suggested that “ESS oil could be used as anti-diabetic complement in case of DM” (p.97). Likewise, Sankar et al [41] specially investigated the efficacy of sesame oil in treating diabetes in human subjects. This study found that rather than using sesame oil treatment alone or orthodox treatment alone, a combination treatment had the greatest effect in ameliorating the side effects of diabetes, with the sesame oil interacting synergistically with standard anti-diabetic medication. Not only does this support the present study’s finding that vegetable and fish oils can have a profound effect in the amelioration of diabetes side effects, it also supports the suggestion that synergistic possibilities exist with respect to the use of these oils in the treatment of diabetes mellitus.

Further, one broader implication of the findings of this study is that there is, in fact, a very significant dietary component to type 2 diabetes mellitus. In the experiment, diet played an enormous role both first in inducing diabetes in the rats and then in ameliorating/preventing the side effects of this induced condition. This general mechanism can be extrapolated to the general structural factors which are presently contributing to the contemporary diabetes epidemic. As Schroder [42] has noted, certain dietary patterns, such as the Mediterranean pattern, confer protection against health problems such as diabetes and obesity. Conversely, obesity is one of the major causes of diabetes, and Caballero [4] has discussed how contemporary society produces conditions which are extremely favorable to the proliferation of an obesity – and by extension, diabetes – epidemic. Such questions are clearly beyond the scope of the present study; but nevertheless, the fact that “natural” chemicals such as vegetable and fish oils can have such dramatic effects on the amelioration of diabetes side effects suggests that there is a significant behavioral component to the rise or mitigation of diabetes within a person or population.

One potential limitation of this study was the relatively small sample size: each group in the experiment, considered in itself, only consisted of 5 rats. This could mean that the evidence supporting the findings pertaining to the specific effects of specific oils may be relatively weak. Future research may wish to narrow the scope of research in order to enhance the power of specific findings: for example, an experiment involving 50 rats and only 2 groups – a single control group and a single experimental group – may be poised to provide more powerful evidence with respect to the very specific question under investigation by that study. Nevertheless, the present study provides an adequate overview of data regarding the question of the efficacy of vegetable and fish oils; and independently from each particular experimental group, the findings incontrovertibly demonstrate the efficacy of an oil diet treatment strategy in general.

In conclusion, the findings of this study strongly support the efficacy of vegetable and fish oils in the amelioration and even prevention of the side effects of type 2 diabetes mellitus. Further research into is warranted; and such research should focus on more clearly identifying the specific properties of specific oils, as well as the possibilities for synergistic interactions not only between the various oils themselves but also between the oils and orthodox anti-diabetic medications.
